# A Quartz Crystal Microbalance Immunosensor for Stem Cell Selection and Extraction

**DOI:** 10.3390/s17122747

**Published:** 2017-11-28

**Authors:** Ornella Maglio, Salvatore Costanzo, Rosaria Cercola, Gerardo Zambrano, Marco Mauro, Raffaele Battaglia, Gianluca Ferrini, Flavia Nastri, Vincenzo Pavone, Angela Lombardi

**Affiliations:** 1 Department of Chemical Sciences, University of Napoli “Federico II” Via Cintia, 80126 Napoli, Italy; ornella.maglio@unina.it (O.M.); salvatore.costanzo@upmc.fr (S.C.); rc1274@york.ac.uk (R.C.); gerardo.zambrano@unina.it (G.Z.); flavia.nastri@unina.it (F.N.); vincenzo.pavone@unina.it (V.P.); 2 Istituto di Biostrutture e Bioimmagini, CNR, Via Mezzocannone 16, 80134 Napoli, Italy; 3 Novaetech S.r.l., Centro Direzionale, Isola G7, 80143 Napoli, Italy; mauro@novaetech.com (M.M.); battaglia@novaetech.com (R.B.); ferrini@novaetech.com (G.F.)

**Keywords:** QCM immunosensor, anti-CD34 antibody, stem cells, self-assembled monolayer, dental pulp

## Abstract

A cost-effective immunosensor for the detection and isolation of dental pulp stem cells (DPSCs) based on a quartz crystal microbalance (QCM) has been developed. The recognition mechanism relies on anti-CD34 antibodies, DPSC-specific monoclonal antibodies that are anchored on the surface of the quartz crystals. Due to its high specificity, real time detection, and low cost, the proposed technology has a promising potential in the field of cell biology, for the simultaneous detection and sorting of stem cells from heterogeneous cell samples. The QCM surface was properly tailored through a biotinylated self-assembled monolayer (SAM). The biotin–avidin interaction was used to immobilize the biotinylated anti-CD34 antibody on the gold-coated quartz crystal. After antibody immobilization, a cellular pellet, with a mixed cell population, was analyzed; the results indicated that the developed QCM immunosensor is highly specific, being able to detect and sort only CD34+ cells. Our study suggests that the proposed technology can detect and efficiently sort any kind of cell from samples with high complexity, being simple, selective, and providing for more convenient and time-saving operations.

## 1. Introduction

During the last few decades, quartz crystal microbalances (QCMs) have become powerful and well-established noninvasive tools for clinical bioassays, and for investigating biomolecular interactions, due to their high sensitivity, low cost, real time output, and label- or radiation-free entities [1,2,3,4,5,6,7,8,9,10]. QCMs are weighting devices based on the piezoelectric properties of the quartz crystal [11,12]. According to Sauerbrey’s equation [13], the frequency shift is linearly proportional to the change of surface mass (Δm) on the crystal:

Δf=(−2f02Aμρq)Δm

where Δ*f* (Hz) is the measured frequency shift; *f*_0_ (Hz) is the fundamental resonance frequency of the quartz oscillator; Δm (g) is the mass change; A (cm^2^) is the active area of the electrode; $\rho_{q}$ is the quartz density; μ is the shear stress of quartz. This proportionality allows for the quantification of the wet adsorbed mass (analyte plus coupled solvent) onto the QCM [14].

The mass sensing of the QCM does not require any labeling step for the signal transduction and enables the measuring of a mass deposition down to 0.1 ng. Furthermore, it is possible to perform measurements in both complex and optically opaque solution media, because the signal transduction mechanism relies upon the piezoelectric effect of crystal quartz [14]. More sophisticated hybrid detection methods have also been developed by integrating QCM with localized surface plasmon resonance (LSPR) [15].

The QCM technique, for its unique advantages and versatility, can be applied to detect a wide range of entities, spanning from small molecules [16,17] to virus and whole cells, such as bacterial and eukaryotic cells [12]. A QCM-based biosensor usually comprises a quartz crystal functionalized with a recognition element, which includes antigens or antibodies, enzymes, or substrates [18,19,20]. The immunoassay format, based on antibody–antigen (Ab–Ag) interactions, remains a robust, ubiquitous analytical tool [21]. Indeed, a number of QCM immunosensors [22] have been reported for the determination of different analytes, such as glucose [4], microbes [23,24,25,26,27], nucleic acids [28,29], environmental pollutants [30], cells [31], and food pathogens [32], for drug discovery applications, and for diagnostics and biomedical applications [33].

In this work, we seek to apply QCM immunosensors in the field of cell biology, for the detection and sorting of stem cells (SCs). Stem cells are unspecialized cells that exhibit self-renewal and multilineage differentiation capability [34,35,36,37]. They are of broad scientific and clinical interest, due to their numerous therapeutic applications. SCs may be used to promote complete tissue repair and avoid detrimental fibrosis, to differentiate and replace damaged cells, providing an alternative to organ transplantation [34]. Further, they have the potential to drastically change the treatment of pathologies such as cancer, Alzheimer's and Parkinson's disease, and paralysis [35]. Among SCs, dental pulp stem cells (DPSCs) are multipotent adult stem cells, expressing mesenchymal and hematopoietic markers [38,39,40]. They are able to differentiate into different tissue of mesenchymal origin, but also in neurons, adipocytes, cardiomyocytes, chondrocytes, melanocytes, and osteoblasts. Thus, DPSCs have shown great potential in regenerative medicine and cell-based therapies for the treatment of various human diseases, including dental problems [38,39].

A drawback for SCs applications is their low occurrence into complex cell mixtures. Thus, a key issue to be addressed is the targeting of specific stem cells. To this end, researchers have been devoting considerable efforts to the development of new methodologies for their detection, isolation, and characterization from different tissues [41,42,43]. Low-resolution methods for stem cell separation exploit their physical parameters, such as size and density, or their ability to adhere onto plastic surfaces. They are widely used for preliminary, fast, and cost-effective separations [42]. Approaches to stem cell separation techniques with high resolution and more specificity involve selection of specific markers, as targets for cell detection and isolation [42,44]. In this respect, immunoaffinity separation techniques, employing antibodies targeting specific cell markers, are the most valuable approaches. By labeling antibodies with fluorescent dyes, instruments such as the fluorescent activated cell sorter (FACS) can be used to detect and isolate target cells. Among flow cytometry [45], FACS technology is widely used for counting and sorting cells tagged with fluorescent probes [46]. The applications of monoclonal antibodies as FACS staining reagents has strongly enhanced the potentiality of one of the most sensitive method for cells isolation [47]. However, FACS application to stem cells has several drawbacks and limitations [42]. The equipment required by this technology is expensive, employs a relatively large sample volume, is difficult to sterilize, is mechanically complex, and can only be operated and maintained by trained personnel.

**Figure 1:**
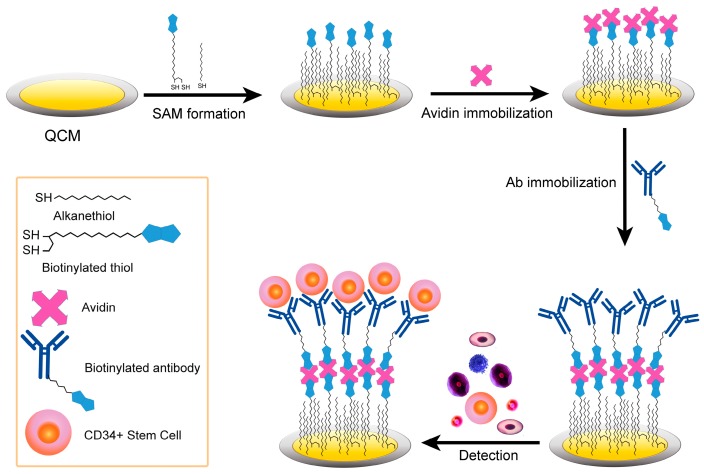
Schematic illustration of the quartz crystal microbalance (QCM) immunosensor for the detection of CD34+ stem cells.

Therefore, alternative methods for stem cell detection and sorting should be developed with the goal of isolating a homogeneous cell population starting from a heterogeneous one. To address this issue, herein we report the development and working principles of a QCM-based immunosensor for the detection and isolation of CD34+ stem cells derived from human dental pulp (see Figure 1).

**Figure 2:**
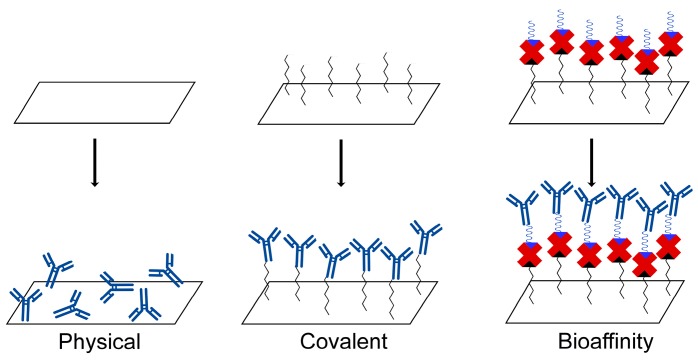
Antibodies immobilization techniques for biosensor construction.

A key aspect of this system is the surface modification of the QCM with antibodies targeting to a specific stem cell marker, the CD34 antigen. Sensitive and reproducible biosensor surfaces need the immobilization of active biomolecules with a controllable and homogeneous orientation, thus retaining their biological activity [48,49,50,51]. This aspect is crucial to antibodies: once immobilized onto a solid support, they could partially lose their specific binding competence, if they are randomly oriented on the surface. To attain ultimate selectivity as well as sensitivity for immunosensing, highly oriented antibody molecular layers on the solid support surfaces are required. Several immobilization techniques have been developed [48,49,50,51], though only three are the most common: physical, covalent, and bioaffinity immobilization (Figure 2).

For immobilizing the antibodies targeting the CD34 antigen on the QCM surface, we selected the bioaffinity technique, based on the biotin–avidin interaction [52,53]. This method is well suited for driving biological interaction and to obtain a well-oriented and stable biomolecule immobilization. To further assure a well-ordered functional monolayer of antibodies, the gold surface of QCM was coated with a mixed biotinylated self-assembled monolayer (SAMs) [54,55,56].

The resulting QCM-based immunosensor allows for the detection of DPSCs and their sorting from a heterogeneous cell population, as confirmed by post-analysis cell culture experiments. The obtained results demonstrate the reliability of the proposed device and its potential applicability in detecting and sorting any cell lineage.

## 2. Materials and Methods

### 2.1. Chemicals and Reagents

Avidin and all chemicals used for the synthesis of biotin-terminated thiol were purchased from Sigma Aldrich, except biotinyl-*N*-hydroxysuccinimide, which was purchased from Novabiochem. Biotinylated anti-CD34 was from tgcBIOMICS. Lyophilized His-tagged-CD34 protein was purchased from Life Technologies. Solvents were purchased from Romil. All solvents and reagents were used without further purification. Phosphate-buffered saline (PBS) and PBS Tween-20 0.05% (*v*/*v*) (PBST) solutions were prepared according to the Cold Spring Harbor protocol with ultrapure water, purchased from Romil. Sulfuric acid 98% and hydrogen peroxide 30% aqueous solutions, used for the cleaning procedure of the QCM gold surfaces, were purchased from Fluka. Pre-coated silica G-60 plates, F254, used for thin-layer chromatography (TLC), were from Merck-Millipore.

The cellular pellet used in this study included a mixed cell population, comprising the 5.6% of stem cells CD34+, as established by cytofluorimetry measurements.

### 2.2. Instrumentation

HPLC and LC-MS analyses were performed with a Shimadzu LC-10ADvp equipped with an SPDM10Avp diode-array detector (Shimadzu Europa GmbH, Duisburg, Germany). ESI-MS spectra were recorded on a Shimadzu LC-MS-2010EV system with ESI interface, Q-array-octapole-quadrupole mass analyzer, and Shimadzu LC-MS solution Workstation software for data processing. For all LC and LC-MS analysis, ultrapure water 0.1% TFA (A) and ultrapure acetonitrile 0.1% TFA (B) were used as solvent system.

The QCM device (open source QCM) was supplied from Novaetech s.r.l. (Napoli, Italy).

ATR experiment was performed on FT-IR Jasco 430 Spectrometer (JASCO Corporation, Tokyo, Japan) utilizing an ATR cell composed by ZnSe, purchased from Pike Technologies (Madison, WI, USA). Spectra were recorded on cleaned quartz crystal at 2 cm^−1^ of resolution with 256 accumulations, from 4000 to 700 cm^−1^.

### 2.3. Synthesis of Biotinylated Linker: N-Dithiooctanoylamidohexyl-5-Biotinylpentanamide (LHB)

**Figure 3:**
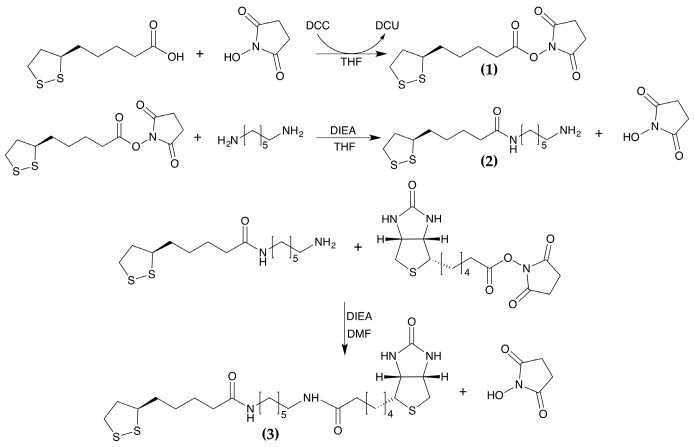
Reaction scheme for the synthesis of the biotinylated linker.

The biotinylated linker is based on lipoic acid (LA) as a thiol anchor, 1,6-hexanediamine (HMDA) as a spacer molecule, and a biotin moiety as a terminal group (Figure 3).

The synthesis was carried out following a stepwise approach.

The lipoic acid was first transformed into its active ester using dicyclohexylcarbodiimide (DCC) and *N*-hydroxysuccinimide (NHS) (Compound **1**). The dithiooctanoyl succinimidyl ester was coupled to 1,6-hexanediamine to provide the mono adduct 6-aminohexyldithiooctanoylamide (Compound **2**).

Finally, the mono adduct was coupled with biotin *N*-succinimidyl ester to obtain the desired product (Compound **3**). The synthesis is described in detail below.

*Dithiooctanoyl succinimidyl ester* (**1**): Lipoic acid (1.0 g, 4.9 mmol) and *N*-hydroxysuccinimide (NHS) (0.56 g, 4.9 mmol) were dissolved in tetrahydrofuran (THF). *N*,*N*′-dicyclohexylcarbodiimide (DCC) (990 mg, 4.9 mmol) was added and the solution was stirred overnight at room temperature. During the reaction course, a white precipitate corresponding to dicyclohexylurea (DCU) was formed. The reaction was monitored by thin layer chromatography (TLC) (eluent: CHCl_3_/CH_3_OH 98/2, Rf = 0.51). The reaction mixture was filtered from DCU, the solution was dried and the compound was crystallized from n-hexane. A yellow powder (1.43 g, yield = 96%), corresponding to the dithiooctanoyl succinimidyl ester was obtained. The ESI-MS spectrum confirmed the molecular weight of the product (theoretical [M-H]^+^: 304.39 *m*/*z*, experimental [M-H]^+^: 304.07 *m*/*z*).

*6-aminohexyldithiooctanoylamide* (**2**): Dithiooctanoyl succinimidyl ester (200 mg, 0.657 mmol) was dissolved in anhydrous THF (10 mL). This solution was added dropwise, under argon, to a solution of HMDA (76.3 mg, 0.657 mmol) in THF (30 mL), and the reaction mixture was stirred at room temperature for 18 h under argon. The reaction was monitored by TLC analysis (eluent: CHCl_3_/CH_3_OH/CH_3_COOH 67/30/3). A white precipitate corresponding to the disubstituted HMDA, containing two terminal lipoic acid units, was observed during the reaction. The reaction mixture was filtered, the solvent was evaporated, and the residue was purified by flash chromatography. The desired product was dried, and its purity was checked by TLC (Rf = 0.57). The product was also analyzed by analytical RP-HPLC-MS (Vydac C18 column, 5% to 40% B over 35 min at a flow rate of 1 mL/min) (theoretical [M-H]^+^: 305.51 *m*/*z*, experimental [M-H]^+^: 305.10 *m*/*z*). The reaction yield was 20% (40.0 mg, 0.131 mmol).

*N-dithiooctanoylamidohexyl-5-biotinylpentanamide (LHB)* (**3**): 6-aminohexyldithiooctanoylamide (40.0 mg, 0.131 mmol)***,*** dissolved in 10.0 mL of DMF, was added to biotinyl-*N*-hydroxy-succinimide (41.9 mg, 0.131 mmol) DMF solution (2.0 mL). The solution was stirred overnight at room temperature. The reaction mixture was analyzed by RP-HPLC-MS. LC-ESI-MS analysis confirmed product formation (theoretical [M-H]^+^: 531.84 *m*/*z*, experimental [M-H]^+^: 531.10 *m*/*z*, experimental [M-Na]^+^: 553.20 *m*/*z*). The crude product was purified by preparative RP-HPLC (Vydac 2.2 cm C18 column, using a 5% to 40% B linear gradient over 35 min, at a flow rate of 22 mL/min). The fractions containing the desired product were pulled together, analyzed by LC-ESI-MS and lyophilized. Pure biotinylated linker was obtained with 70% yield (48.7 mg).

### 2.4. QCM Setup and Detection Test

The quartz crystals (supplied from IEV s.r.l., Varese, Italy) are AT-cut with a fundamental frequency of 10 MHz. The quartz crystal and the gold electrode are 8 and 4.5 mm in diameter, respectively. For all the experiments only one side of the crystal was coated.

Before all experiments, the gold surface was thoroughly cleaned by immersing the quartz crystal in a hot piranha solution (3:1 concentrated H_2_SO_4_/30% H_2_O_2_) for 30 min, rinsed in ultrapure water, followed by ethanol washing. Finally, the quartzes were dried under argon steam.

The gold–quartz crystal was placed on the electronic console and the resonance frequency of the oscillator was monitored. The volume of the liquid interacting with the QCM (i.e., the cell compartment) was approximately 20 μL. The response of the QCM is proportional to the mass tethered to the electrode [11]. The mass sensitivity of the crystal used in this work was 4.41 ng/mm^2^ per 1 Hz of frequency shift.

#### 2.4.1. QCM Immunosensor Assembly

To functionalize the gold surface with a self-assembled thiol mixed monolayer, cleaned quartz crystals were left in contact with a solution containing 0.01 mM LHB and 1 mM 11-MUA (ratio 1/100) for 18 h at room temperature. The quartzes were then rinsed with ethanol and water and dried under argon steam.

Biotin-derivatized quartz crystals were installed into the QCM detection chamber. The whole detection procedure was monitored in real time by the QCM biosensor and the frequency changes of the crystals were recorded. The oscillation frequency of the quartz crystals was monitored until stable (10 min) after 100 μL of phosphate buffer were infused.

Afterwards, 20 μL of avidin (5 mg/mL in phosphate buffer pH 7.4) were incubated onto the crystal until saturation was reached. The resonance frequency, during the avidin addition was recorded in real time and no change in frequency was observed after 20 min. Subsequently, the crystals were rinsed with the washing solution (PBS pH 7.4 with 0.05% Tween 20, PBST) to remove any un-specifically bound molecule. A 30 μL solution of BSA-PBS solution (25 μg/mL) was added to cover any un-filled space on the gold surface and avoid possible non-specific interactions in further steps. Finally, 20 μL of biotinylated anti-CD34 antibodies (25 μg/mL in PBS pH 7.4) were directly placed onto the functionalized gold surface, until no variation in oscillation frequency was observed. The excess of antibody was removed by PBS and PBST washing.

#### 2.4.2. Procedure for the Detection of DPSCs

The detection of stem cells derived from dental pulp was performed as follows: 20 μL of the cellular pellet, composed of 500,000 cells/mL (5.6% was constituted by stem cells CD34+), was introduced with a syringe into the QCM cell and the frequency shift of the sensor was monitored for 30 min until it plateaued.

In order to reduce the exposure time of the stem cells to mechanical vibration of the quartz, the immunoassay was performed adding the stem cells to the switched-off QCM device. After 30 min of incubation, the frequency shift was measured. After the measurement, the liquid flow was switched back to the PBS buffer until a stable baseline was obtained, to wash out bound analyte and prepare the crystal for further use.

After the analysis, the quartz was incubated into a culture medium to verify the cellular growth.

## 3. Results and Discussion

### 3.1. Derivatization of the Gold Surface for QCM Immunosensor Construction

In order to immobilize anti-CD34 specific monoclonal antibodies for DPSC detection, the gold surface was modified via a mixed SAM strategy. Bare gold surface functionalization was performed using thiolated linkers, exploiting the strong affinity between gold and sulfur [54,55,56,57].

A crucial issue in the development of the QCM immunosensor was the establishment of an optimal biotin density on the gold surface. Previous studies, performed on different kinds of SAM, showed that good control over the surface density of biotin groups is extremely important and that a loosely packed monolayer is needed to afford a stable avidin monolayer for the upcoming antibody immobilization [49]. A loosely packed mixed SAM was afforded by mixing 11-MUA and a LHB. The biotinylated linker designed and developed in this work is based on lipoic acid as a thiol anchor. It provides two sulfur atoms that ensure a tight and secure binding onto gold surface. 1,6-Hexanediamine was chosen as a spacer molecule between LA and biotin, which was used for affinity interaction with the avidin (Figure 3). According to literature, a 100:1 ratio of 11-MUA/LHB was used to obtain a moderate biotin coverage of the SAM surface [58].

Fuctionalized gold surfaces were characterized by attenuated total reflectance infrared spectroscopy (ATR-FTIR). The presence of the peaks at 2930 cm^−1^ and 2858 cm^−1^, assigned to the ν_as_(CH_2_) and ν_s_ (CH_2_) modes of methylene groups, respectively, can be ascribed to the 11-MUA and LHB alkyl chains. The vibrational pattern of stretching signals was not characterized by sharp peaks. This phenomenon can be attributed to the presence of different morphological domains onto the surface of polycrystalline gold. Furthermore, the bands at 1520–1660 cm^−1^ are consistent with amide vibrations modes and provide reliable evidence that biotin molecules are bound onto the surface [59].

### 3.2. Immobilization of Anti-CD34 Antibodies on QCM Surface and Test for CD34 Protein Binding

**Figure 4:**
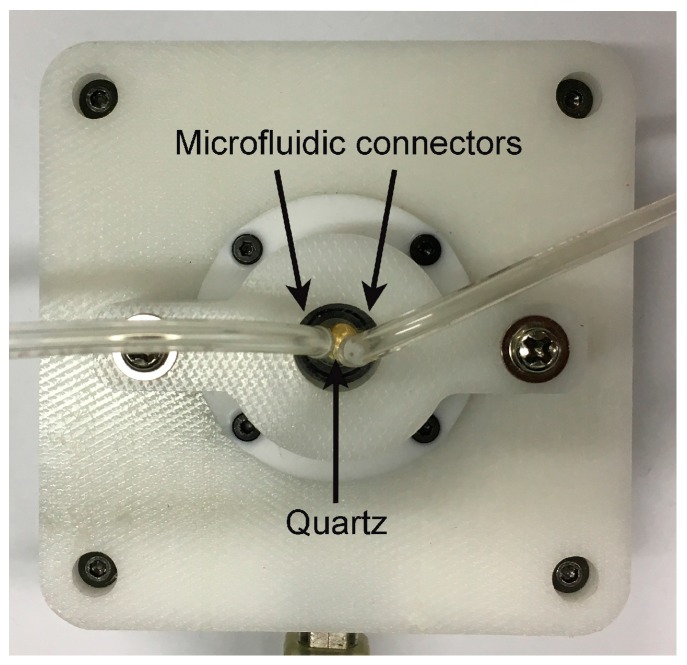
Flow cell of the QCM device manufactured by Novaetech s.r.l.

The mixed SAM-coated quartz crystals were used to immobilize anti-CD34 antibodies. Figure 4 shows the flow cell of the QCM device.

**Figure 5:**
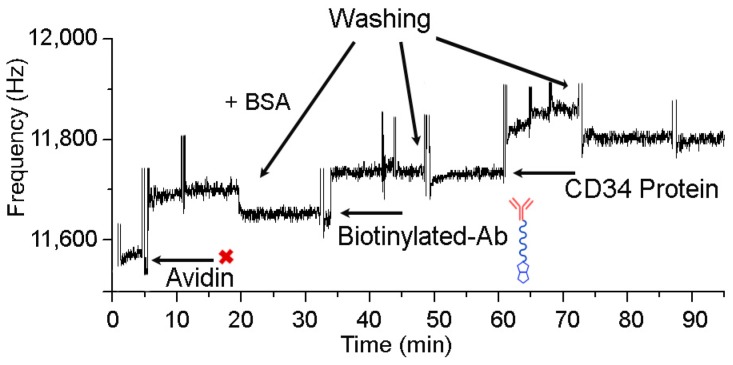
Frequency shift mesurements in the QCM immunosensor for the CD34 protein detection.

To assay the functionality of the immobilized antibodies and to evaluate the reliability of the QCM device in a bio-sensing application, a test for CD34 protein binding was performed, after immobilization of the anti-CD34 antibodies onto the avidin layer. Figure 5 reports the real-time sensor resonance frequency shift, acquired during this experiment.

It should be noted that the frequency shift corresponds to the beat frequency of the sensor compared with the reference crystal. The QCM device used in this work provides a more accurate estimation of frequency shift by comparing each measurement to a reference passive crystal. The difference between the sensor and the reference crystal produces curves with a positive profile. This asset minimizes variations between measurements, thus ensuring reproducibility in replicates.

In the first step, avidin at a concentration of 5 mg/mL in phosphate buffer was added to the biotin-derivatized crystals until saturation was reached. A frequency change confirmed the biotin-avidin recognition event (Figure 5) [60,61]. The avidin immobilization showed high reproducibility: ΔF values ≈ 115 Hz were measured in several experiments carried out on different crystals (data not shown).

After avidin immobilization, the crystals were rinsed with the washing solution, and subsequently 20 µL of 25 μg/mL BSA solution were added. The addition of BSA solution did not determine any frequency shift, thus indicating the complete surface coverage and the presence of a homogeneous layer of avidin. Biotinylated anti-CD34 antibodies were then added to the flow cell and their immobilization was ascertained by a frequency shift of 70 Hz; saturation was reached after 20 min.

After antibody immobilization, a solution of His-tagged-CD34 protein (20 µL, 25 μg/mL) was added and a frequency shift of 60 Hz was observed. These results confirmed the specificity of the avidin/biotinylated Ab system and the possibility to use this system to detect stem cells.

### 3.3. Stem Cell Detection by QCM-Immunosensor

**Figure 6:**
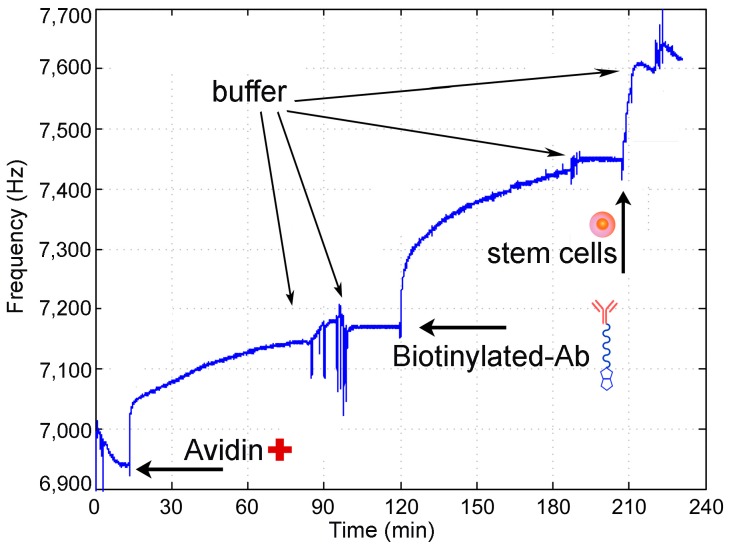
Frequency shift measurements in the QCM immunosensor for stem cell detection.

A typical assay for stem cells detection consisted of the same steps reported in Section 3.2, with the differences that a mixture of cells, instead of His-tagged-CD34 protein, was added to the QCM-immunosensor in the last step. The assay steps can be summarized as follows: (1) frequency measurement of the biotin-functionalized quartz crystal; (2) measurement of frequency shift after avidin addition; (3) measurement of frequency shift after anti-CD34 biotinylated antibody binding; (4) measurement of frequency shift upon stem cell sample addition. The frequency shift observed in each step of the assay is reported in Figure 6.

After addition of the avidin solution to the flow cell containing biotinylated gold surface, saturation was reached in about 90 min. Avidin adsorption caused a frequency shift of 207 Hz. Once avidin was immobilized on the surface, the crystal was washed with PBS and Tween 20 solution. Then, biotinylated anti-CD34 antibody was added and the resonance frequency of quartz crystal was monitored for about 70 min until saturation was reached. The crystal was rinsed with the washing solution, to remove the unbound or weakly adsorbed antibodies and a frequency shift of 261 Hz was observed.

As shown by the Sauerbrey equation [13], there is a linear correlation between the resonant frequency of the quartz crystal and the mass of a substance deposited on its surface. This correlation is valid when a thin rigid film is added on the crystal surface; the film should be uniform (constant density and thickness), and cover the acoustically active area of the whole crystal. The behavior of a quartz crystal is different in liquid phase, where the response frequency of the QCM also depends on the density and the viscosity of the contacting media (the frequency shift from air to aqueous solution is in the range 4–12 kHz). For this reason, in the liquid-phase, a term containing the viscosity (η_L_) and the density (ρ_L_) of the solution should be taken into account. Nevertheless, as reported by Wang et al. [26], in the static liquid-phase measurement, the viscosity and the density of the background buffer are constant in each measurement, so their contribution can be overlooked. Based on these considerations, the mass change and the amounts of molecules of each layer on the gold surface were calculated using the following equation [26]:

Δm=−Δf(AC f02) where C= 2μρq.

Considering a quartz density of 2.648 g cm^−3^ and a shear stress for AT-cut quartz of 2.947 × 10^11^ g cm^−1^ s^−2^, the C value is 2.26 × 10^−6^ cm^2^ Hz^−1^ g^−1^. The measured frequency shifts, the calculated adsorbed mass, and number of molecules in each layer are reported in Table 1.

Inspection of Table 1 indicates that the QCM-based immunosensor is capable of binding up to 10^12^ molecules of the target analyte. However, this amount can be affected by several different factors, such as antibody activity, temperature, and pH. The results herein reported are in good agreement with those described for QCM-based immunosensors developed for *Escherichia coli* O157:H7 detection [26,27].

After the antibody immobilization, the sample containing stem cells was added to the detection cell. The sample consisted of a cellular pellet with a heterogeneous cell population, comprising the 5.6% of stem cells CD34+, as established by cytofluorimetry measurements. Twenty microliters of this suspension were added to the QCM cell, and the frequency was monitored for 30 min until saturation was reached: the observed ΔF was of 165 Hz (Figure 6).

This result indicates that molecular recognition occurs and that the designed immunosensor can be successful used to detect stem cells.

The experiment for stem cells detection was carried out in triplicate. In order to assess stem cell detection reproducibility, a statistical analysis on three independent replicas was performed. A data set of 500 sample was extracted for each replica, corresponding to each step of the detection assay. The possibility of combining data sets coming from different replicas is justified under the hypothesis that each experimental test has been performed in the same condition (i.e., measurement apparatus and setup, concentration of chemical species, etc.); thus, the frequency measurements are influenced only by random errors. However, data analysis may not be trivial, so a more detailed statistical analysis was carried out.

**Figure 7:**
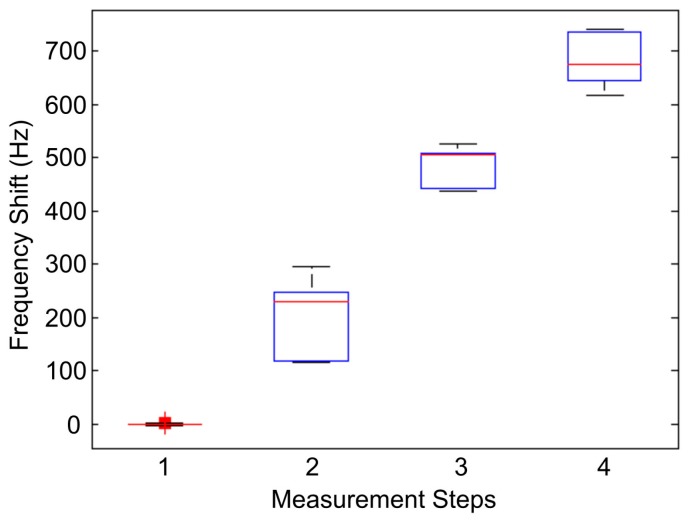
Box plot related to the four steps of data set collection. The measurement steps correspond to (1) determination of zero, (2) avidin, (3) antibody, and (4) stem cells. Each box shows the median (red), 25th and 75th percentile (blue borders), and maximum and minimum (bases in black).

Statistical populations are shown in Figure 7 using a box plot to display variation for each data set. Average frequency shift values obtained from the three independent replicas are reported in Table 2.

Firstly, the statistical populations do not show outliers. The starting frequency measurement was stable, demonstrating the device reproducibility. Populations corresponding to avidin addition and antibody binding showed a higher dispersion for values below the mean value, while the frequency variation corresponding to stem cells had a more symmetric behavior around the expected value. Value dispersion in Steps 2 and 3 might have occurred since proteins can pack themselves under a variety of possible arrangements onto the gold surface, thus originating SAMs with variable density. Step 4, instead, exhibited a more uniform distribution of points around the mean value. The higher homogeneity could have been because, upon cells binding, a large number of antibodies become inaccessible due to the ligand hindrance. Therefore, the differences in antibody packing did not influence the overall binding capacity of the QCM immunosensor, ensuring high reproducibility in sensor construction and reliability in sensor measurements.

All these results indicate the efficacy of the designed immunosensor in successfully detecting and sorting CD34+ cells, such as DPSCs. Real-time detection of living cells by QCMs has already been reported [62], as well as the ability of QCM devices to assess cellular adhesion, differentiation processes, self-renewal activity, and cell–substrate interactions [41,43]. It should be pointed out that QCM immunosensors have not been applied for stem cell detection and sorting, to the best of our knowledge [41]. Furthermore, the methodology herein proposed takes advantages of two strong binding: the biotin–avidin recognition has been combined with the antigen–antibody interaction, the most selective molecular recognition known in biochemistry.

As outlined in the introduction, the most widely used methods for living cell detection and sorting, such as FACS [46,47], actually count cells passing through the detector. The proposed method provides a cost-effective, time-saving alternative by avoiding the use of expensive fluorescently labeled antibodies and flow cytometers. Moreover, the integration of the QCM immunosensor, hereby developed, in the microfluidic system remarkably scales down the amount of sample needed. Even though QCM devices measure wet mass adsorbed onto the crystal, this aspect does not influence the overall potentiality of the technique, when compared to FACS. Indeed, QCM-based sensors can appreciate very small differences in wet mass adsorbed, down to 0.1 ng (compared with putative cell mass weight of 3.5 ng [63]).

All these findings broaden the QCM applications in SC research and pave the way to the development of fully customizable protocols for living cell detection in unknown samples and their direct sorting, applicable in both diagnostic fields [64] and cell biology [41].

**Figure 8:**
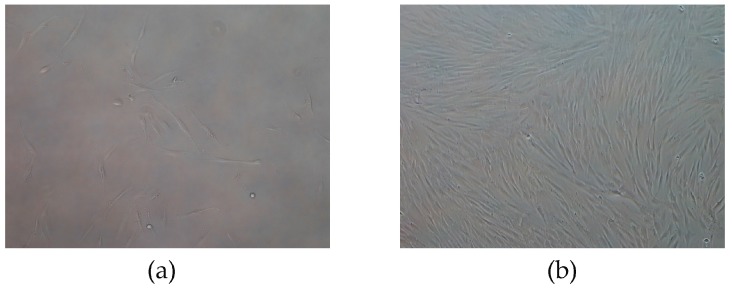
(**a**) Cells after 24 h of culture (original magnification × 100). (**b**) Cells at confluence after 1 week of culture (original magnification × 100).

To verify that the proposed detection technique did not alter stem cell behaviors, an experiment of cell growth on the quartz crystal immunosensor was performed. The quartz crystal was rinsed with the washing solution, placed into the cell culture plate, and treated with trypsin/EDTA, in order to detach the cells from its surface. The cells were then filtered and immersed in Dulbecco’s Modified Eagle Medium (DMEM) 10% FBS culture medium for 7 days at 37 °C in 5% CO_2_. Visual inspections after 24 h revealed cell growth, which further increased after seven days (Figure 8).

In order to assess the nature of the grown cells, osteogenic differentiation experiments were carried out (alpha-MEM buffer 20% FBS for 30 days) and proved that all the detected cells were CD34+ stem cells. These results confirmed the validity of the developed QCM-based immunosensor in detecting and sorting stem cells, without affecting their differentiation capacities.

## 4. Conclusions

An effective biosensor for the direct detection and sorting of dental pulp stem cells (DPSCs) has been developed. It combines QCM technology and the SAM-based strategy for antibody immobilization onto gold surfaces. The formation of SAMs offers great versatility in terms of bio-recognition, thus allowing reliable control over packing density. Surface functionalization was obtained by coating the gold surface of QCM, through a mixed SAM composed by thiolated chains with and without end-attached biotin groups. This layer has been used for specific avidin immobilization that acts as a cross-linker between the sensor surface and the anti-CD34 antibodies. Using this asset, the developed QCM-based immunosensor was able to discriminate CD34+ stem cells within a heterogeneous cellular sample. As ascertained by cell culture and osteogenic differentiation experiments, the detection and sorting method did not alter stem cell differentiation potentials. All the results obtained strongly suggest that the proposed QCM biosensor is a selective, fast, and economic method for the detection and sorting of stem cells. By using different kinds of stem cell markers, the proposed methodology could be applied to different stem cells if proper antibodies are selected. Furthermore, the proposed QCM device holds several advantages: simple technology in production, extension to a variety of biomarkers, possibility of miniaturization, ease of use, and low cost. As a consequence, such a device may have great potential with regard to point-of-care (POC) testing for early, reliable, and non-invasive detection of important diseases [64].

## Figures and Tables

**Table 1:** Frequency shift, mass, and number of molecules in each step of surface functionalization.

Layer	MW (Da)	Δ*f* (Hz)	Δ*m* (ng)	mol (pmol)	N
Avidin	66,000	207	145	2.20	1.32 × 10^12^
Antibody	150,000	261	184	1.23	0.74 × 10^12^

**Table 2:** Average frequency shift values obtained from the three independent replicas.

	Starting Frequency	Avidin	Anti-CD34	Stem Cell
Average value (Hz)	0	203	485	684
Median (Hz)	−0.12	229	506	676
Standard Deviation (Hz)	1.2	62	32	40
Mean Absolute Deviation (Hz)	1.3	56	30	34
